# Wind Wave Behavior in Fetch and Depth Limited Estuaries

**DOI:** 10.1038/srep40654

**Published:** 2017-01-18

**Authors:** Arash Karimpour, Qin Chen, Robert R. Twilley

**Affiliations:** 1 Louisiana Sea Grant, Louisiana State University, Baton Rouge, LA 70803, USA; 2 Department of Civil and Environmental Engineering, Louisiana State University, Baton Rouge, LA 70803, USA; 3 Center for Computation and Technology, Louisiana State University, Baton Rouge, LA 70803, USA; 4 Coastal Studies Institute, Louisiana State University, Baton Rouge, LA 70803, USA; 5 Department of Oceanography and Coastal Sciences, Louisiana Sea Grant, and Coastal Studies Institute Louisiana State University, Baton Rouge, LA 70803, USA

## Abstract

Wetland dominated estuaries serve as one of the most productive natural ecosystems through their ecological, economic and cultural services, such as nursery grounds for fisheries, nutrient sequestration, and ecotourism. The ongoing deterioration of wetland ecosystems in many shallow estuaries raises concerns about the contributing erosive processes and their roles in restraining coastal restoration efforts. Given the combination of wetlands and shallow bays as landscape components that determine the function of estuaries, successful restoration strategies require knowledge of wind wave behavior in fetch and depth limited water as a critical design feature. We experimentally evaluate physics of wind wave growth in fetch and depth limited estuaries. We demonstrate that wave growth rate in shallow estuaries is a function of wind fetch to water depth ratio, which helps to develop a new set of parametric wave growth equations. We find that the final stage of wave growth in shallow estuaries can be presented by a product of water depth and wave number, whereby their product approaches 1.363 as either depth or wave energy increases. Suggested wave growth equations and their asymptotic constraints establish the magnitude of wave forces acting on wetland erosion that must be included in ecosystem restoration design.

As one of the most productive natural ecosystems in the world, wetland dominated estuaries provide biological, ecological, economic and cultural services to the environment and surrounding human communities^1^. The many benefits of estuaries include nursery grounds for fisheries, habitat for migratory birds, and nutrient filters for improved water quality, which consequently contribute to the economy through commercial fishing, tourism, and recreational activities^1^,^2^,^3^. The growing risks of wetland loss in these coastal ecosystems jeopardize their significant ecosystem services, demanding for more intense efforts to protect and restore these coastal landscapes^4^,^5^,^6^. A critical feature of such restoration projects is to understand how erosion may contribute to wetland loss in these shallow environments, where depth and fetch are perceived to limit wave generation. There is increased awareness that even in shallow estuaries dominated by wetland vegetation, the wave activity contributes to processes such as sediment re-suspension, mudflat erosion, turbidity alteration, marsh edge erosion and wetland losses^7^,^8^,^9^,^10^.

Previous studies suggested that wind wave activities in wetland dominated estuaries are a potential factor in enhancing and accelerating wetland loss rates^10^,^11^, which fundamentally can affect the physics and biology of estuaries. The conversion of wetlands to water caused by wind waves in shallow estuaries, leads to further increases in wind fetch, which consequently causes wave generation with higher energy. Over time, wetland loss generates a positive feedback between increased fetch and more energetic wave generations, which increase wetland erosion. Shallow estuaries are categorized as coastal water bodies with a short wind fetch and shallow to intermediate water depth, limiting the physical aspects that contribute to wave generation and growth in these environments. Wetland loss causes variations in these physical aspects and consequently in wave generation, requiring new analytics to fully capture wind wave behavior under these conditions prior to any ecosystem restoration design.

The study of wave predictions goes back to the Beaufort wind scale, which aimed at the qualitative description of wind forcing and wave height. Since then, wave generation and growth have been studied in diverse environments to predict wave properties in both deep and depth limited water. The SMB method^12^,^13^,^14^ was one of the earliest wave models, followed later by more accurate studies such as JONSWAP in deep water^15^, TMA in depth limited water^16^, and recent studies in fetch limited shallow water^17^,^18^,^19^,^20^. Although parametric wave prediction models have been presented for depth and fetch limited conditions, the physical aspects of wave growth in these environments are not fully understood and require further studies. The main goal of our study is to expand the knowledge of wind wave growth in depth and fetch limited water and to improve wave prediction methodology under these conditions.

Our experimental design includes a field study in Breton Sound (BS Dataset) and Terrebonne Bay (TB Dataset), both in Louisiana, USA, along with re-analysis of the existing datasets from Lake George, Australia (YV96 Dataset^17^, YB06 Dataset^19^). Wave generation is governed by a limited set of parameters, such as wind fetch, water depth, wind velocity and bottom friction. Although Lake George is a closed inland lake while estuaries are typically semi-enclosed water bodies, owing to the similarity of the governing parameters in terms of wave generation, the field data collected at those sites are used in the present study. Both field study sites in the USA are located on the northern coast of the Gulf of Mexico (Fig. 1). The first site was in Breton Sound at 29°31′46.26″*N* and 89°24′42.24″*W*, with wind fetch ranges from 14.8 × 10^3^ *m* to 86.7 × 10^3^. The second site was in Terrebonne Bay at 29°11′13.20″*N* and 90°36′33.59″*W*, with wind fetch ranges from 3.3 × 10^3^ *m* to 36.4 × 10^3^ *m*. Data were collected between November 13 and December 22, 2009 in Breton Sound, and between August 24 and December 31, 2010 in Terrebonne Bay. Both bays have experienced reduced area of wetlands and barrier islands over the last century, demonstrating changes in fetch limitation as these wetland dominated estuaries deteriorate. In fact, between 1932 and 2010, Breton Sound and Terrebonne Bay lost 451 × 10^6^ *m*^2^ and 1309 × 10^6^ *m*^2^ wetland area, respectively, the latter representing the largest land loss rate in Louisiana^21^.

Assimilation and collective analysis of the four datasets, two from field studies in Louisiana and two from re-analysis of existing datasets in Australia, clearly revealed the ultimate limit for wave growth in depth-limited water. Commonly, the asymptotic limits for wave growth are presented as a function of dimensionless water depth, 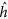, but it was shown that using dimensionless peak wave number, 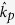, might be a better alternative to present 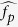 asymptotic limit^19^. Directed by these two approaches, we discovered that the asymptotic limits for wave growth in shallow water can be properly presented by illustrating 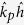 as function of dimensionless water depth, 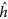, and dimensionless wave energy, 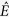 (see methods section for variable descriptions). Then, the asymptotic limit equations are selected and fitted to the edge of the dataset as:


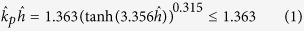



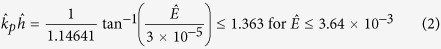


The asymptotic limits of 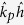, which represent the longest probable wind waves that can be obtained in depth limited water, are defined by eqs (1) and (2) as functions of 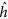 and 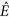, respectively (Fig. 2a,b). With respect to 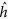, the asymptotic limit of 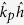, i.e. eq. (1), first increases rapidly as 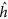 increases, but its slope diminishes until it ultimately becomes independent of 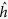 for 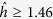, where it becomes constant at 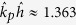. Similarly, the asymptotic limit of 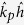 with respect to 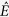, i.e. eq. (2), first increases rapidly with 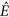, but as 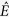 increases further, 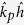 becomes independent of 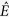 and eventually approaches 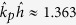. The threshold of 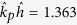 is well established for Stokes’ wave modulation^22^,^23^,^24^,^25^. Although 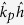 asymptotic limits become independent of the 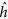 or 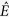 as either depth or wave energy increases, both 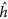 and 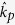 remain variable along the asymptotic limit lines resulting from eqs (1) and (2). In summary, when 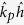 approaches 1.363 and becomes constant, the 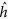 and 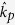 do not become constant along those asymptotic limit lines.

Conventionally, the asymptotic limits are presented by dimensionless peak wave frequency, 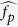, and dimensionless wave energy, 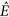, as a function of dimensionless water depth, 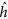. Therefore, to find the asymptotic limit of 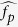 as a function of 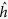, the values calculated from eq. (1) are solved along with the dispersion relationship, i.e. (2*πf*_*p*_)^2^ = *gk*_*p*_ tanh (*k*_*p*_*h*). Similarly, the asymptotic limit of 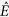 as a function of 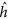, is found by rearranging eq. (2) into 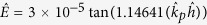 and solving it along with eq. (1). Following these methods, the approximated solutions for the asymptotic limits of 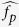 and 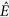 are:


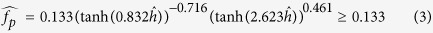



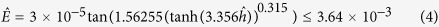


The values of 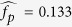 and 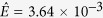 are associated with the fully developed condition^26^. In contrast to the existing methods, the new approach of using 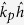 asymptotic limits to develop asymptotic limits of 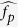 and 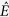 as a function of 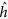, provides a smooth transition of the asymptotic lines towards the fully developed condition (Fig. 2c,d).

Wave growth in a fetch limited, deep water environment is well accepted to be a function of wind fetch and wind velocity, and is presented with dimensionless fetch, 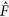, in power law forms of 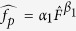 and 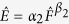
18. There were enormous experimental efforts devoted to defining the coefficients *α* and *β* for various situations, which resulted in a wide range of empirical values for both *α* and *β*^27^,^28^,^29^, with some attempts to relate *β*_1_ and *β*_2_^28^,^29^.

In addition to wind fetch and wind velocity, water depth plays an important role in shallow and intermediate water wave growth. Under this condition, wave growth is often presented as a function of 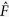 and 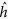
17,30, which mostly does not conform to a power law. A later study on wave growth in shallow estuary led to two major findings^31^. First, it was shown that the 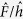 ratio is an important factor in 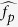 prediction in shallow water, and the implementation of this ratio allows to present the 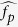 in a power law form. Second, the exponent *β*_1_ is not constant and varies as a function of the 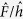 ratio. Using the 4 datasets in this study, we are able to demonstrate that in fact both 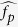 and 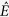 in shallow and intermediate water are functions of 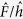 (Fig. 3a,b), and can be presented in a power law form as:


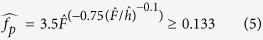



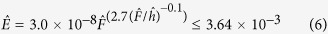


Both exponents *β*_1_ and *β*_2_ in eqs (5) and (6) are function of 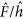, which indicates that the wave growth rate in depth limited water is controlled by the 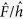 ratio (Fig. 3c,d). Combining eqs (5) and (6), the relationship between 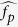 and 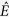 would be 
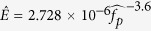
.

Results from eqs (5) and (6) need to be limited to the fully developed condition, i.e. 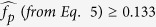 and 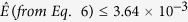, to the depth limited water asymptotic limits, i.e. 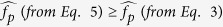 and 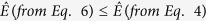, and to the values for 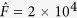 when 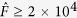, i.e. 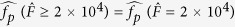 and 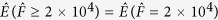. Depending on the water body properties, results from eqs (5)) and (6) might need to be limited to the deep water wind wave growth rates such as JONSWAP^15^, i.e. 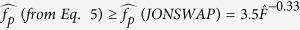 and 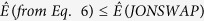 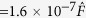.

In order to meet the fetch limited condition, the duration of a sustained wind should be long enough to allow waves to travel the entire fetch distance of *F*. Using a mean depth averaged along the fetch *F*, a minimum duration of the sustained wind required for the depth limited water waves to be considered fetch limited can be expressed in a dimensionless form as:


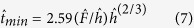


The upper limit of eq. (7) is determined by 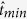 value from either deep water or fully developed condition, whichever is smaller. One recommendation is 
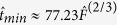
 for the deep water^32^ and *gt*_*min*_/*U*_*A*_ = 7.15 × 10^4^ where 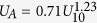 for the fully developed condition^30^. If the dimensionless sustained wind duration is less than the minimum duration of 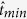, then the wave growth is considered as a duration-limited. In this case, an equivalent wind fetch is calculated and used in eqs (5) and (6). To calculate an equivalent wind fetch, the 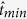 in eq. (7) is replaced by a dimensionless sustained wind duration and is solved for 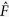. Depending on the water body properties and wind condition, a full fetch instead of the equivalent fetch might need to be used even in a duration-limited condition. For instance, rapid changes in wind direction result in the duration-limited wave growth. To account for the pre-existing energy in the area caused by the rapid wind rotation, a full wind fetch instead of the equivalent fetch might need to be used.

The new approach presented here for developing the asymptotic limits of wave growth based on 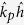 values, helps to accurately define the asymptotic limits of peak wave frequency and wave energy in depth limited water, with a smooth transition to the fully developed condition. This improves our understanding of energy build-up and transfer during the final stage of wave growth, as it reveals that asymptotic 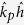 values approach 1.363 and become independent of the 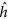 or 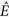 as either depth or wave energy increases. The dependency of the 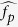 and 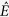 on the ratio of the wind fetch to water depth, 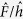, helps to develop a new set of parametric wave growth equations for depth and fetch limited environments. Furthermore, it reveals that the wave growth rate in a depth limited water is not constant and is a function of 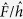. This ratio leads to the development of a new criterion to define if waves are fetch limited.

Clarification of how fetch and depth influence wave generation is a critical element of estuarine dynamics in wetland dominated estuaries, such as deltaic coasts and other sediment rich coastal regions around the world. As wetland loss occurs from complex interactions of sediment supply, subsidence and sea-level rise in estuaries with significant total area occupied by wetlands, the fetch limited wave functions become an important component of accelerated erosional force on wetland landscapes^10^. Fetch enlargements due to wetland loss lead to a wave generation with higher energy and consequently to a higher rate in wetland erosion. This accelerated wetland loss caused by a positive feedback among wetland erosion, fetch increases and more energetic waves generation, changes the ecosystem services in wetland dominated estuaries. Therefore, better analytics in how fetch limited waves behave in depth limited estuaries will be a critical part of designing estuary restoration projects. Suggested asymptotic constraints on wave generation in shallow estuaries establish the magnitude of wave forces acting on wetland erosion that must be included in ecosystem restoration design. The proposed wave growth methods can support a new, convenient and practical means for an accurate prediction of the wind waves in such fetch and depth limited environments.

## Methods

### Non-dimensional parameters

The dimensionless values for the peak wave frequency, 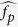, wave energy, 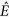, water depth, 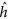, wind fetch, 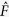, peak wave number, 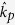, and minimum duration of the sustained wind required for wave to become fetch limited, 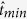, all denoted by ^ symbol are defined as^12^:


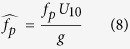



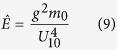



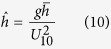



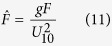



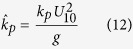



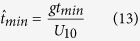


where *g* is the gravitational acceleration, *m*_0_ is the zero-moment or area under the water surface elevation power spectral density (see wave analysis section for descriptions), *U*_10_ is a 10-minute averaged wind velocity at a height of 10 *m* from surface, *F* is the wind fetch, and 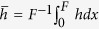 is the mean water depth averaged over the length of the wind fetch, *h* is a local water depth, *x* is distance along the fetch axis, *f*_*p*_ is the peak wave frequency, *k*_*p*_ is the wave number associated with the peak wave frequency, and 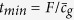 is the minimum time required in second for the wave to travel the distance of *F* where 
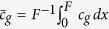
 is a wave mean group velocity along the fetch axis, and *c*_*g*_ is a wave group velocity.

### Data collection

Pressure and velocity measurements were carried out at 0.8 *m* and 1.09 *m* above the seabed, respectively, by deploying a bottom-mounted Acoustic Doppler Velocimeter (ADV) on the sea floor in an up-looking reading mode. Data were recorded for 1024 seconds in 30-minute intervals at 2 *Hz* (Terrebonne Bay) and 4 *Hz* (Breton Sound) sampling frequencies. The 10-minute average wind data at 10 *m* above the surface level were obtained from the National Oceanic and Atmospheric Administration (NOAA) station at Shell Beach, LA (SHBL1), located at 29°52′5″*N* and 89°40′24″*W*, for Breton Sound and from the LUMCON monitoring station located adjacent to the ADV deployment location in Terrebonne Bay.

### Wind data evaluation

After all wind data were adjusted to reflect the velocity at 10 *m* above the surface level, they were evaluated for being sustained and steady in both magnitude and direction. They were considered steady if both 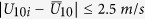 and 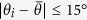 were met, where *U*_10*i*_ and *θ*_*i*_ are wind velocity and wind direction at the *i*^*th*^ data point, respectively, and 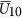 and 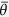 are the mean values of wind velocity and wind direction averaged over the preceding consecutive data points which consecutively satisfied the steady state conditions, respectively^31^,^32^. Then, all duration-limited wind data were defined as if the sustained wind duration in second was less than the minimum duration of 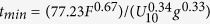 and excluded from the dataset^31^,^32^.

### Wave analysis

Total spectral wave energy was calculated from 
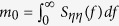
, where 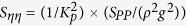 is the water surface elevation power spectral density, *S*_*PP*_ is the dynamic pressure power spectral density, *K*_*p*_ = cosh(*kd*_*p*_)/cosh(*kh*) is the dynamic pressure to the surface elevation conversion factor, *ρ* is the density of water, *k* is the wave number and *d*_*p*_ is the pressure measurements’ distance from the bed. Peak wave frequency was acquired from the surface elevation spectrum’s peak^31^.

### Swell energy removal

The power spectra from the Breton Sound and Terrebonne datasets were examined for the presence of the swell energy from the Gulf of Mexico through the openings between degraded barrier islands, and in case of the swell presence, the swell energy was removed from the spectrum following the spectrum sea-swell partitioning method^31^,^33^. Furthermore, an inverse wave age, 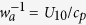 where *w*_*a*_ is the wave age and *c*_*p*_ = *g*/(2*πf*_*p*_) is a phase speed of the peak wave, was calculated and only the sea state waves with *U*_10_/*c*_*p*_ > 0.83 were retained in the datasets^31^.

### Datasets

Breton Sound dataset (BS Dataset) and Terrebonne dataset (TB Dataset) contain 1855 and 6200 measurement points, respectively, each point represents a 30-minute burst. Based on aforementioned criteria, 243 and 468 data points were retained in BS Dataset and TB Dataset, respectively, for this study. Existent datasets from Lake George, Australia, consist of 994 data points in YV96 Dataset^17^, all in north-south direction, and 92 data points in YB06 Dataset^19^, with no fetch data reported in YB06 Dataset.

### Existing models

The Shore Protection Manual^30^ suggested following equations for the wave properties prediction in the fetch and depth limited water^31^:


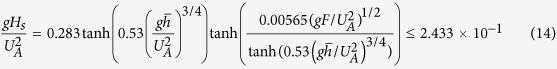



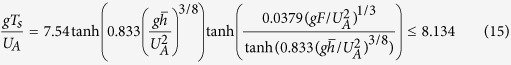



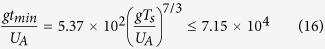


where 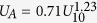 is an adjusted wind velocity, *H*_*s*_ ≈ *H*_*m*0_ is a significant wave height, 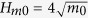 is a zero-moment wave height, *T*_*s*_ ≈ 0.95*T*_*p*_ is a significant wave period, and *T*_*p*_ = 1/*f*_*p*_ is a peak wave period. The values 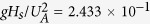, *gT*_*s*_/*U*_*A*_ = 8.134 and *gt*_*min*_/*U*_*A*_ = 7.15 × 10^4^ are associated with the fully developed condition. Based on the observations in Lake George, Australia, Young and Verhagen^17^ modified the Shore Protection Manual^30^ equations for the fetch and depth limited water as^31^:


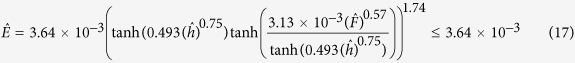



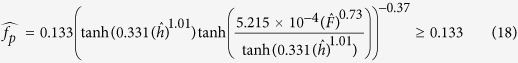


Young and Verhagen^17^ suggested asymptotic limits in fetch and depth limited water as 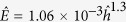 and 
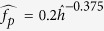
, which the former one was modified by Young and Babanin^19^ as 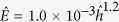.

### Model performance assessment

The accuracy of the proposed asymptotic limits to predict the edge of the dataset is illustrated in the supplementary information. Furthermore, the accuracy of the proposed wave growth model is evaluated through the assessment of the goodness of fit using the root-mean-square error, *RMSE*, scatter index, *SI*, Nash–Sutcliffe efficiency coefficient, *NSE*, Pearson’s correlation coefficient, *r*, coefficient of determination, *R*^2^, and normalized mean bias, *NMB*. Results of the new parametric model performance compared to the existing models are presented in detail in the supplementary information.

## Additional Information

**How to cite this article**: Karimpour, A. *et al*. Wind Wave Behavior in Fetch and Depth Limited Estuaries. *Sci. Rep.* **7**, 40654; doi: 10.1038/srep40654 (2017).

**Publisher's note:** Springer Nature remains neutral with regard to jurisdictional claims in published maps and institutional affiliations.

## Supplementary Material

Supplementary Information

## Figures and Tables

**Figure 1:**
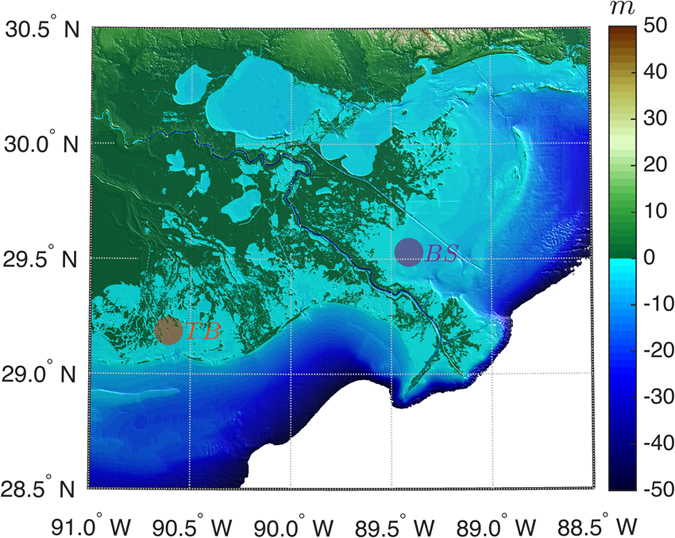
Bathymetry of study area. Breton Sound site and Terrebonne Bay site are marked with *BS* and *TB*, respectively. Data are from NOAAs’ USA Coastal Relief Model (www.ngdc.noaa.gov/mgg/coastal/crm.html) and NOAAs’ VDatum Digital Elevation Model (DEM) Project (http://www.ngdc.noaa.gov/mgg/inundation/vdatum/vdatum.html). Map is generated using MATLAB R2014b (www.mathworks.com). The color bar represents elevation in *m*.

**Figure 2:**
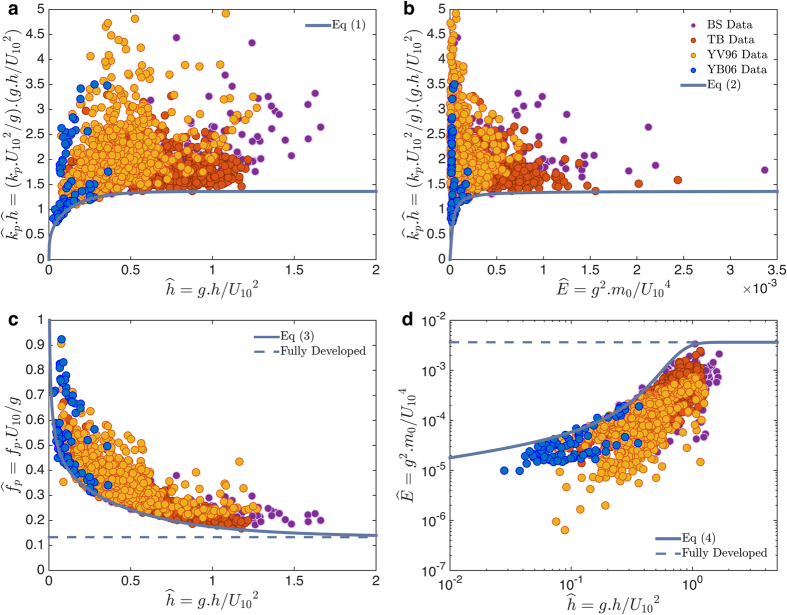
Wave growth asymptotic limits. (**a**,**b**) The smallest 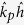 that wind waves can grow in fetch limited shallow waters as a function of 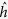 (**a**) and 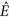 (**b**), respectively. (**c**,**d**) the asymptotic limits for 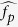 and 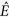 as a function of 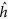, respectively. The horizontal dashed-line represents the fully developed condition.

**Figure 3:**
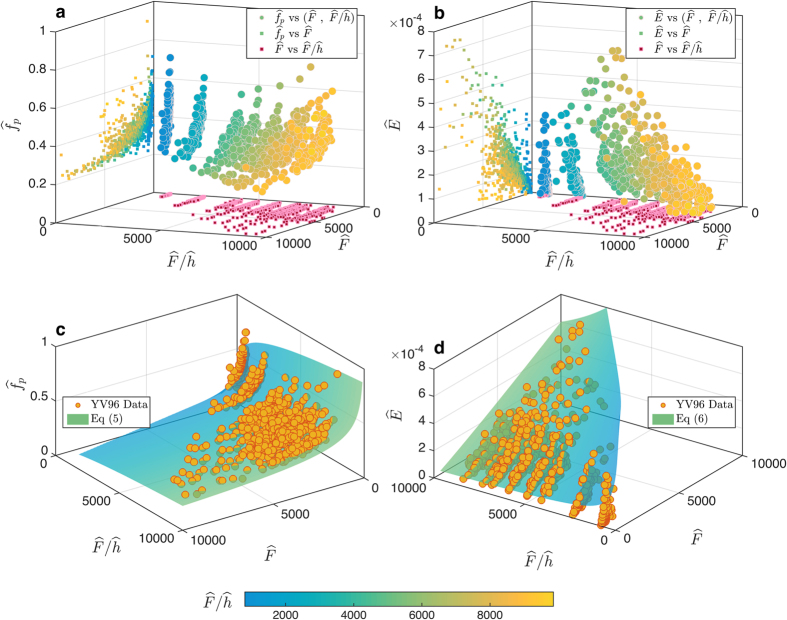
Wave growth in fetch limited shallow water. (**a**,**b**) Variation of the 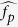 and 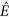 as function of 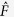 and 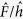. Dependency of 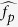 and 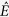 on 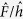 are presented both in 3D (circle markers) and 2D (square markers) plots. (**c**,**d**) Eqs (5) and (6) are plotted against the YV96 Dataset. The color bar represents 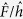.
