# Supplementary material for: Wind Wave Behavior in Fetch and Depth Limited Estuaries
Source: Sci Rep. 2017 Jan 18;7:40654. doi: 10.1038/srep40654 (PMC5241670; doi:10.1038/srep40654)
Supplement: Supplementary Information [file srep40654-s1.pdf]

Wind Wave Behavior in Fetch and Depth Limited Estuaries
Supplementary Information

Arash Karimpour[1]*, Qin Chen[2,3,4] and Robert R. Twilley[4,5]

Model performance assessment. Proposed asymptotic limits for dimensionless peak wave frequency and wave energy along with existing asymptotic limits for depth limited water are illustrated in Figure 4. The proposed equations (3) and (4) accurately define the edge of the dataset while smoothly transit toward the fully developed condition.

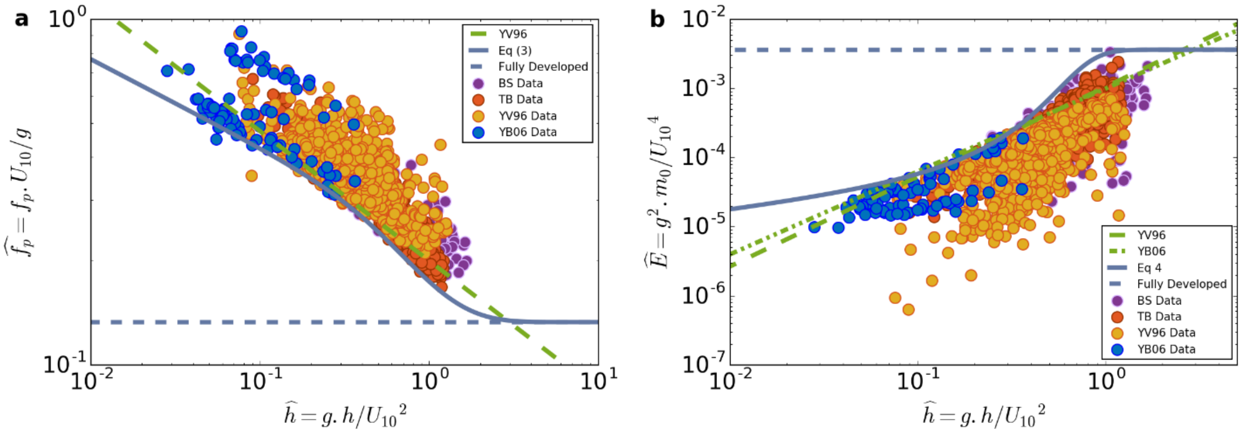

Figure 4 | Asymptotic limits. The asymptotic limit equations (3) and (4) along with existing asymptotic limits for depth limited water from Young and Verhagen[17] (YV96) and Young and Babanin[19] (YB06) are presented. **a**, Dimensionless peak wave frequency. **b**, Dimensionless wave energy. The horizontal dashed-line represents the fully developed condition. Plot is generated by Matplotlib[34].

The accuracy of the proposed wave growth model is evaluated through the assessment of the goodness of fit using the root-mean-square error, $RMSE$, scatter index, SI, Nash–Sutcliffe efficiency coefficient, NSE, Pearson's correlation coefficient, r, coefficient of determination, R^2, and normalized mean bias, NMB, using BS, TB and YV96 datasets consist of $N = 1705$ paired samples of (X, Y), where X and Y are observed and estimated values, respectively, as:

[1] Louisiana Sea Grant, Louisiana State University, Baton Rouge, LA 70803, USA
[2] Department of Civil and Environmental Engineering, Louisiana State University, Baton Rouge, LA 70803, USA

[3] Center for Computation and Technology, Louisiana State University, Baton Rouge, LA 70803, USA

[4] Coastal Studies Institute, Louisiana State University, Baton Rouge, LA 70803, USA

[5] Department of Oceanography and Coastal Sciences, Louisiana Sea Grant, and Coastal Studies Institute Louisiana State University, Baton Rouge, LA 70803, USA

*e-mail: akarimp@g.clemson.edu

$$RMSE = \sqrt{\frac{\sum(Y_i - X_i)^2}{N}} \quad (19)$$

$$SI = \frac{RMSE}{\bar{X}} \quad (20)$$

$$NSE = 1 - \frac{\sum(Y_i - X_i)^2}{\sum(X_i - \bar{X})^2} \quad (21)$$

$$r = \frac{\sum(X_i - \bar{X})(Y_i - \bar{Y})}{\sqrt{\sum(X_i - \bar{X})^2 \sum(Y_i - \bar{Y})^2}} \quad (22)$$

$$R^2 = r^2 = \left(\frac{\sum(X_i - \bar{X})(Y_i - \bar{Y})}{\sqrt{\sum(X_i - \bar{X})^2 \sum(Y_i - \bar{Y})^2}}\right)^2 \quad (23)$$

$$NMB = \frac{\bar{Y} - \bar{X}}{\bar{X}} \quad (24)$$

The root-mean-square error, $RMSE$, provides the differences between the observed variable and estimated variable[31]. The scatter index, SI, is defined as $RMSE$ normalized by mean of the observed values and shows dispersion respect to the line with a slope of 1:1. The Nash–Sutcliffe efficiency coefficient, NSE, indicates how well observed and estimated data fit the line with a slope of 1:1. It ranges from $-\infty$ to 1, where $NSE = 1$ corresponds to a perfect match between observed and estimated data, $NSE = 0$ indicates that the predictions are as accurate as the mean of the observed data, and $NSE < 0$ shows that the mean of observed values is a better predictor than the estimated ones[31]. The Pearson's correlation coefficient, r, represents the degree of the linear correlation between observed and estimated values. It ranges between -1 and 1, where $r = 1$ stands for complete positive correlation, $r = 0$ for no correlation, and $r = -1$ for complete negative correlation[31]. The coefficient of determination, R^2, indicates how well model fits the data. It returns a value between 0 and 1, where $R^2 = 1$ indicates model perfectly fits the data, and $R^2 = 0$ indicates that model does not fit the data at all. The normalized mean bias, NMB, defines an average tendency of the estimated results compared to the observed values, where for positive observed and estimated values, $NMB = 0$ indicates identical means for observed and estimated values, $NMB > 0$ shows a model has an overestimation bias, and $NMB < 0$ indicates a model has an underestimation bias.

Using these coefficients, the goodness of fit for the proposed model in this study and previous models are presented in Tables 1 and 2, representing the new parametric model performance compared to the existing models.

Table 1 | $\widehat{f_p}$ estimation accuracy.

	Goodness of fit for $\widehat{f_p}$					
**Method**	$RMSE$	SI	NSE	r	R^2	NMB
Shore Protection Manual[30], Eq. 15	0.0571	0.1756	0.5674	0.7824	0.6122	-0.0121
Young and Verhagen[17], Eq. 18	0.0503	0.1547	0.6644	0.8279	0.6855	-0.0334
This Study, Eq. 5	0.0495	0.1523	0.6749	0.8585	0.7371	-0.0603

Table 2 | $\widehat{E}$ estimation accuracy.

	Goodness of fit for $\widehat{E}$					
**Method**	$\boldsymbol{RMSE}$	$\boldsymbol{SI}$	$\boldsymbol{NSE}$	$\boldsymbol{r}$	$\boldsymbol{R^2}$	$\boldsymbol{NMB}$
Shore Protection Manual[30], Eq. 14	2.31×10^{-4}	0.8917	0.3057	0.6568	0.4314	0.2691
Young and Verhagen[17], Eq. 17	2.06×10^{-4}	0.7953	0.4478	0.6736	0.4537	0.0541
This Study, Eq. 6	1.97×10^{-4}	0.7581	0.4983	0.7115	0.5062	0.0188

References:

17. Young, I. R. & Verhagen, L. A. The growth of fetch limited waves in water of finite depth. Part 1. Total energy and peak frequency. *Coastal Engineering*, 29, 47-78 (1996).

19. Young, I. R. & Babanin, A. V. The form of the asymptotic depth-limited wind wave frequency spectrum. *Journal of Geophysical Research*, 111 (C06031) (2006).

30. Department of the Army, Waterways Experiment Station, Corps of Engineers, and Coastal Engineering Research Center, Shore Protection Manual, Washington, D.C., vol. 1, 4th ed., 532 pp (1984).

31. Karimpour, A. & Chen, Q. A Simplified Parametric Model for Fetch-Limited Peak Wave Frequency in Shallow Estuaries. *Journal of Coastal Research*, 32(4), 954-965 (2016).

34. Hunter, J. D. Matplotlib: A 2D graphics environment. *Computing in science and engineering*, 9(3), 90-95 (2007).
